# Entomopathogenic fungi (Hypocreales: Ophiocordycipitaceae) infecting bark lice (Psocoptera) in Dominican and Baltic amber

**DOI:** 10.1080/21501203.2019.1706657

**Published:** 2019-12-22

**Authors:** George Poinar, Fernando E. Vega

**Affiliations:** a Department of Integrative Biology, Oregon State University, Corvallis, OR, USA; b Sustainable Perennial Crops Laboratory, U. S. Department of Agriculture, Agricultural Research Service, Beltsville, MD, USA

**Keywords:** Fossil fungus, Dominican and Baltic amber, bark lice, Psocoptera, Hypocreales

## Abstract

*Ophiocordyceps dominicanus* Poinar & Vega sp. nov. in Dominican amber and *Polycephalomyces baltica* Poinar & Vega sp. nov. (Hypocreales: Ophiocordycipitaceae) in Baltic amber are described as entomopathogenic fungi of bark lice (Psocoptera). The specimens possess several features unknown in extant synnematous entomopathogenic fungi such as a tubular dark synnema with a straight, pointed tip bearing spores over the entire surface in *O. dominicanus*, and a globular yellowish synnema developing on the tip of the host’s antenna in *P. baltica*. These are the only known fossil entomopathogenic fungi of bark lice, making them unique not only for their characters but also in respect to their selection of developmental sites on their bark lice hosts.

## Introduction

A number of fungi, including some pathogenic on insects and spiders, have conidiophores united into synnemata (large erect reproductive structures bearing compact conidiophores, phialides and conidia) (Kirk et al. 2001). These synnemata can have long stalks arising from the body of their hosts or the stalk can be quite reduced and with the fertile terminal portion sphere-shaped. Some lineages are restricted to just one or a few host species, or even to specific tissues on a host, while others appear to be able to parasitise a range of hosts, even in different orders (Barnett 1958; Kendrick and Carmichael 1973; Poinar and Thomas 1984).

The present study describes two new species of fossil fungi of the order Hypocreales and family Ophiocordycipitaceae that formed synnemata on bark lice (Psocoptera) in Dominican and Baltic amber, respectively. These are the only known entomopathogenic fungi of fossil bark lice. Even extant records of infection of bark lice by members of the Hypocreales are limited to one account of a species of *Hirsutella* infecting a bark louse in Argentina (Toledo et al. 2008).

## Materials and methods

The Dominican Republic amber piece containing the infected bark louse measures 12 mm long, 8 mm wide, and 3 mm deep. The specimen originated from mines in the El Mamey Formation (Upper Eocene) in the Cordillera Septentrional of the Dominican Republic. These mines consist of shale-sandstone and rounded pebbles (Eberle et al. 1980) and their age has been estimated at 15–20 Ma (Iturralde-Vinent and MacPhee 1996) and 20–23 Ma (Baroni Urbani and Saunders 1980) based on foraminifera counts, to 30–45 Ma based on coccoliths (Schlee 1990). These are considered minimum dates since they are based on microfossils in the strata containing the amber, which was secondarily deposited in turbiditic sandstones of the Upper Eocene to Lower Miocene Mamey Group (Draper et al. 1994). The location of the amber mines is DD 19.5 N, 70.6 W.

Fossiliferous amber from the Baltic region was formed in forests occurring in what have been described either as tropical/subtropical climates or humid warm-temperate climates that covered a large part of northern Europe for some 10 million years during the early Palaeogene (Sadowski et al. 2017). The amber was eroded from the original accumulation and re-deposited in marine sediment layers referred to as the ‘blue earth' layers, in the Samland Peninsula of the Russian Republic (Weitschat and Wichard 2010). Age estimates of amber from this region range from 35 to 55 million years (Larsson 1978; Wolfe et al. 2016).

A Nikon SMZ-10 R stereoscopic microscope (Nikon Instruments, Tokyo) and Nikon Optiphot compound microscope (Nikon Instruments, Tokyo) with magnifications up to 800 X were used for observing and photographing the specimens. Photos were stacked using Helicon Focus Pro X64 (HeliconSoft, Kharkiv, Ukraine) to improve clarity and depth of field.

Both amber pieces were polished close enough to the surface of the synnemata to observe the spores under oil immersion. The following descriptions are based on features observed in different regions of the synnemata.

## Results

### Dominican amber specimen

#### Taxonomy

Kindgom FungiPhylum: AscomycotaOrder: HypocrealesFamily: OphiocordycipitaceaeGenus: *Ophiocordyceps*

**Figure 1.**
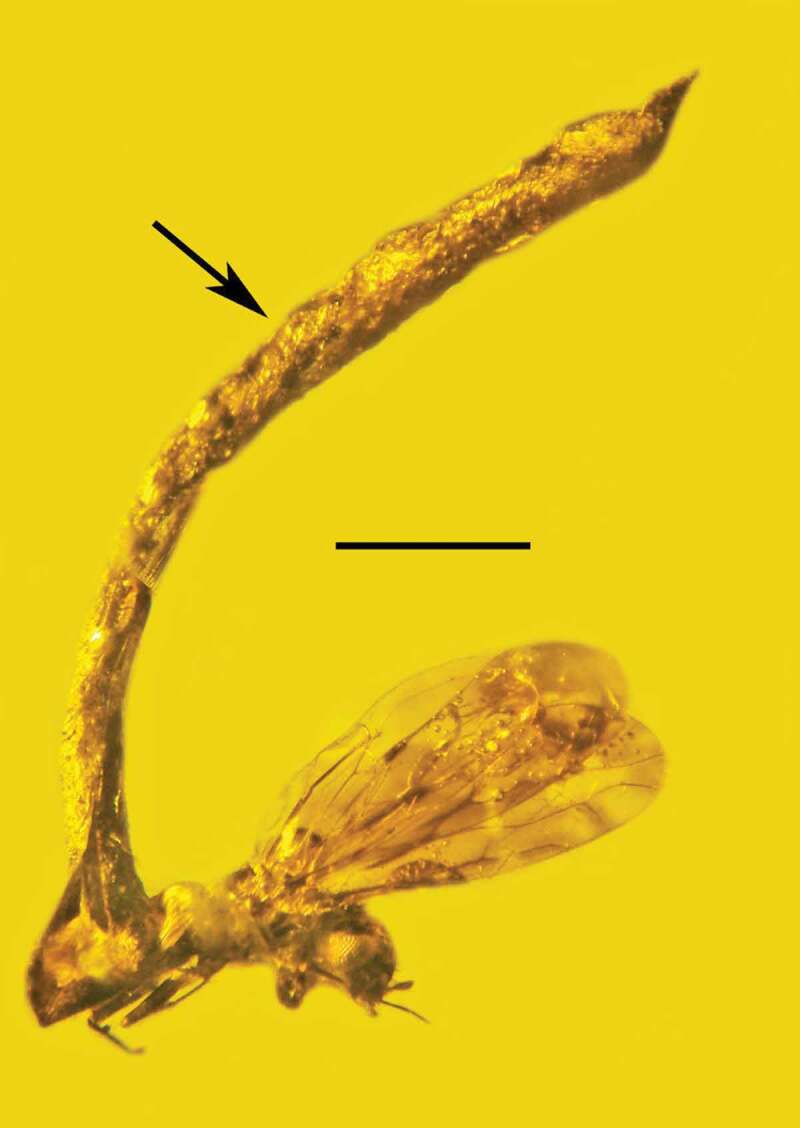
Synnema (arrow) of *Ophiocordyceps dominicanus* sp. nov. attached to the body of a bark louse (Psocoptera: Troctopsocidae) in Dominican amber. Bar = 0.6 mm.

**Figure 2.**
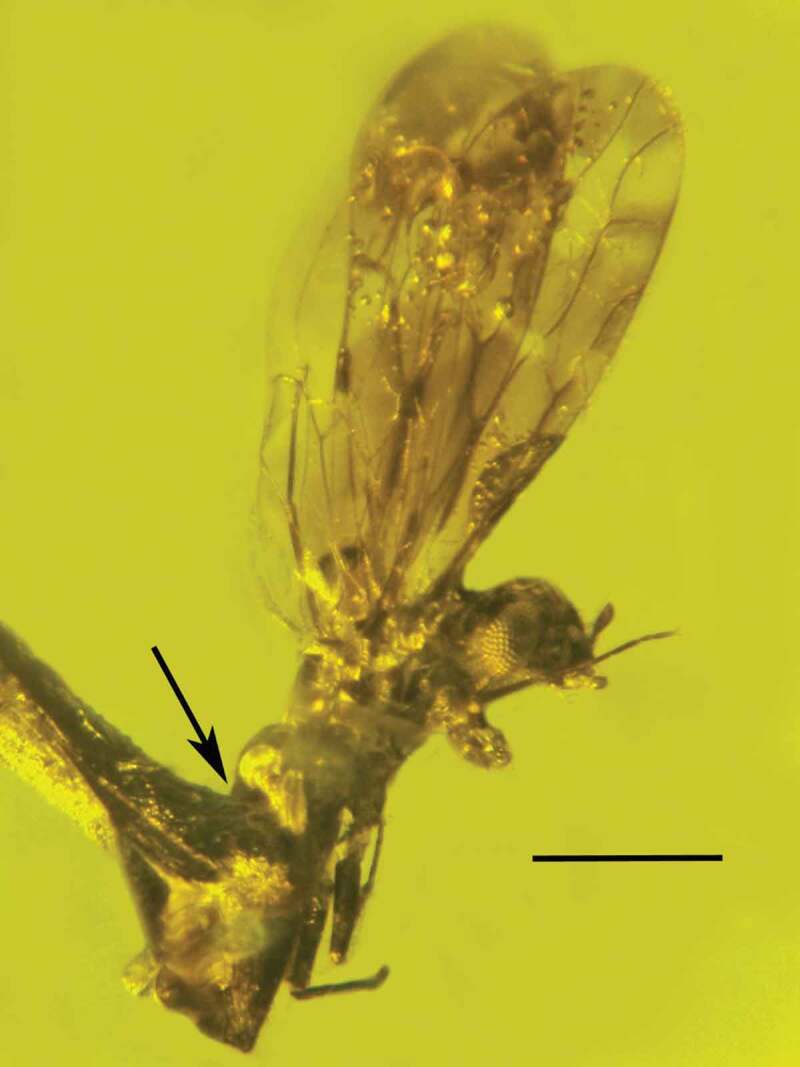
Attachment point (arrow) of the synnema of *Ophiocordyceps dominicanus* sp. nov. to the body of a bark louse (Psocoptera: Troctopsocidae) in Dominican amber. Bar = 0.3 mm.

**Figure 3.**
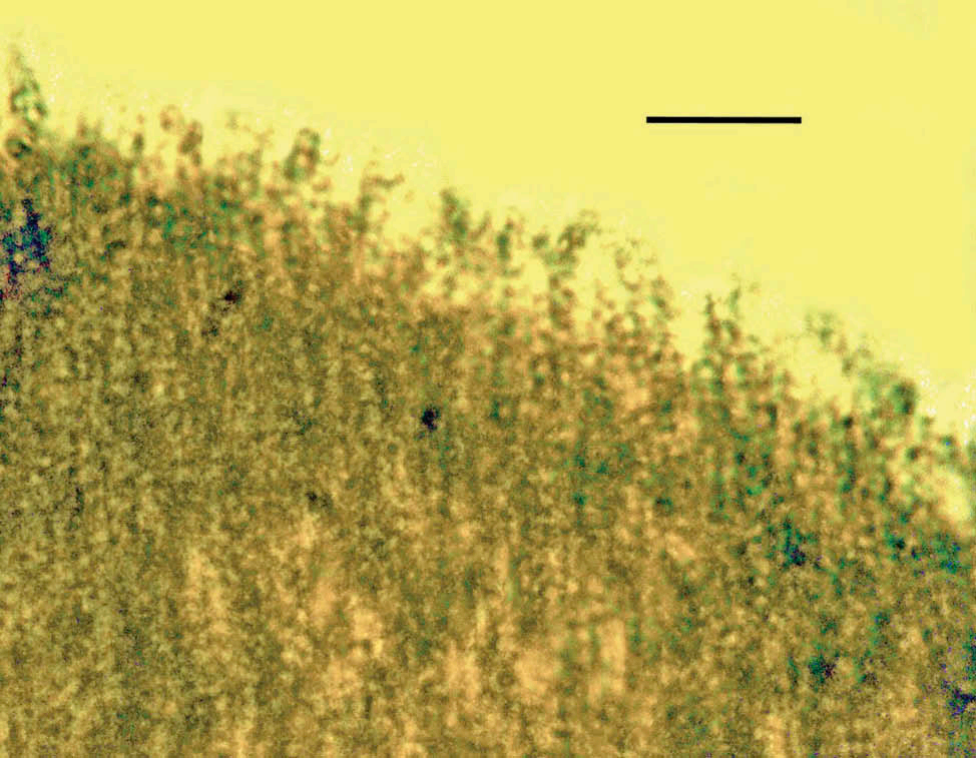
Surface of synnema covered with spores of *Ophiocordyceps dominicanus* sp. nov. in Dominican amber. Bar = 12 µm.

**Figure 4.**
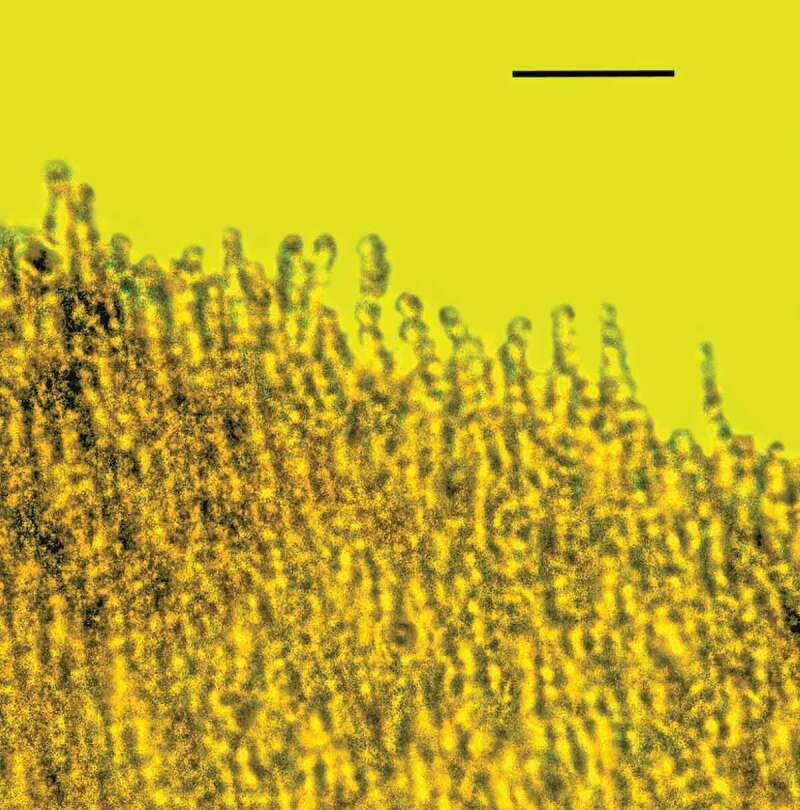
Chains of conidia of *Ophiocordyceps dominicanus* sp. nov. arising from surface of synnema in Dominican amber. Bar = 14 µm.

**Figure 5.**
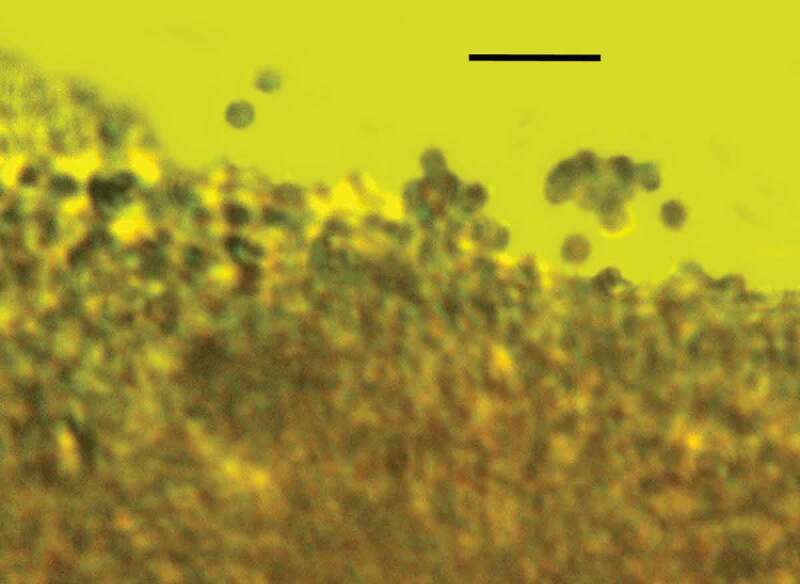
Loose spores of *Ophiocordyceps dominicanus* sp. nov. released from chains in Dominican amber. Bar = 10 µm.

**Figure 6.**
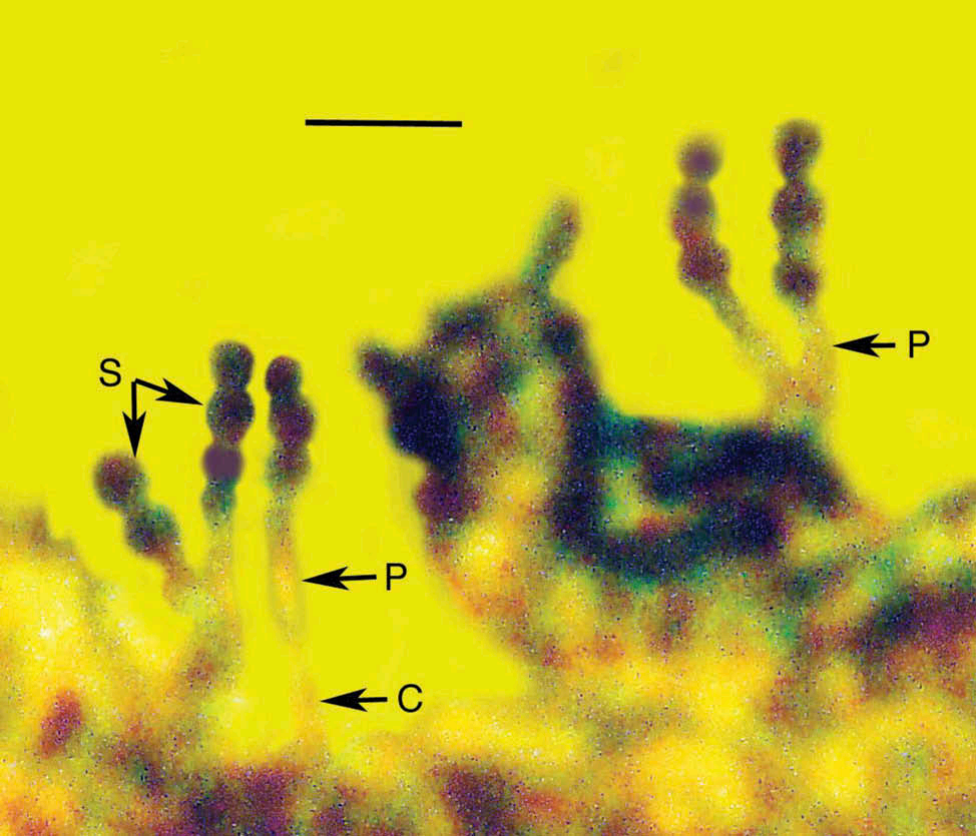
Detail of conidiophore (C), phialide (P) and conidia (S) of *Ophiocordyceps dominicanus* sp. nov. in Dominican amber. Bar = 8 µm.

Type species: *Ophiocordyceps dominicanus* Poinar & Vega sp. nov.*MycoBank*: 830,883*Ophiocordyceps dominicanus* Poinar & Vega sp. nov. (Figures 1–6).

#### Diagnosis

Synnema dark, pointed at tip; entire surface covered with unbranched chains of conidia or released conidia; conidiophores micronematous, phialides micronematous, non-diverging; conidia mostly brown or tan, globose, lacking surface ornamentation, formation basipetal.

#### Description

Synnema simple, 3.5 mm in length, emerging from abdomen of adult bark louse (Psocoptera). Aerial hyphae very rare; entire surface of synnema covered with catenulate or released small, roundish, dark conidia; conidiogenous cells phialiform; conidiophores light coloured, micronematous, length 6–7 µm; phialides light coloured, micronematous, narrow, slender, nondiverging, length, 7–9 µm; conidia globose, one-celled, brown to tan, lacking surface ornamentation or mucus; basocatenate in unbranched spore-chains (with up to 10 spores in a chain), diameter of conidia, 2–3 µm.

##### Holotype

Holotype No. Sy-1-13A deposited in the Poinar amber collection maintained at Oregon State University.

#### Host

Adult *Troctopsocopsis* sp. (length 1.1 mm) (Psocoptera: Troctopsocidae)

#### Type locality

Amber mine in Altamira faces of the El Mamey Formation in the Cordillera Septentrional of the Dominican Republic. DD latitude and longitude: 19.4, −70.4.

#### Comments

The Dominican amber specimen is tentatively assigned to the genus *Ophiocordyceps* at this time, with the realisation that it may be transferred to other genera at a later date, depending on future studies on the Ophiocordycipitaceae. *Ophiocordyceps dominicanus* is unique among entomopathogenic fungi by possessing a straight, pointed synnema bearing spores over the entire surface, micronematous conidiophores and non-diverging phialides bearing unbranched chains of basipetal, globose, brown or tan conidia lacking surface ornamentation or mucus.

*Ophiocordyceps dominicanus* shares some features with other earlier recorded entomopathogenic fungi in the Cordycipitaceae and Ophiocordycipitaceae. One of these is the genus *Gibellula* Cav. (Cordycipitaceae). However, species of this genus have the apex of the conidiophores swollen, forming vesicles that produce pro-phialides, which in turn, form flask-shaped phialides bearing elliptical spores. The entire reproductive unit is globose or wedge-shaped. In most other arthropod-infecting genera, the phialides are not borne in heads. These include members of the genera *Hymenostilbe* Petch (Ophiocordycipitaceae) that produces single, hyaline, elongate conidia on phialides with short lateral branches, and *Akanthomyces* Leb. (Cordycipitaceae) with light coloured synnemata and hyaline, one-celled, ovate conidia borne on wedge-shaped phialides. The genus *Isaria* Pers. ex Fr. (Cordycipitaceae) has light coloured synnemata and hyaline, one-celled, clustered, ovoid conidia. The genus *Hirsutella* Pat. (Ophiocordycipitaceae) has hyaline one-celled mucus-covered conidia borne singly and the phialides are swollen at the base. The genus *Pseudogibellula* Samson & Evans (Cordycipitaceae) has hyaline conidia borne in globose heads at the tips of conidiophores with enlarged apices (Barnett 1958; Kendrick and Carmichael 1973; Poinar and Thomas 1984). None of the features found in the above-mentioned extant genera match those of the fossil.

## Baltic amber specimen

### Taxonomy

Kingdom Fungi Phylum: AscomycotaOrder: HypocrealesFamily: OphiocordycipitaceaeGenus: *Polycephalomyces*

**Figure 7.**
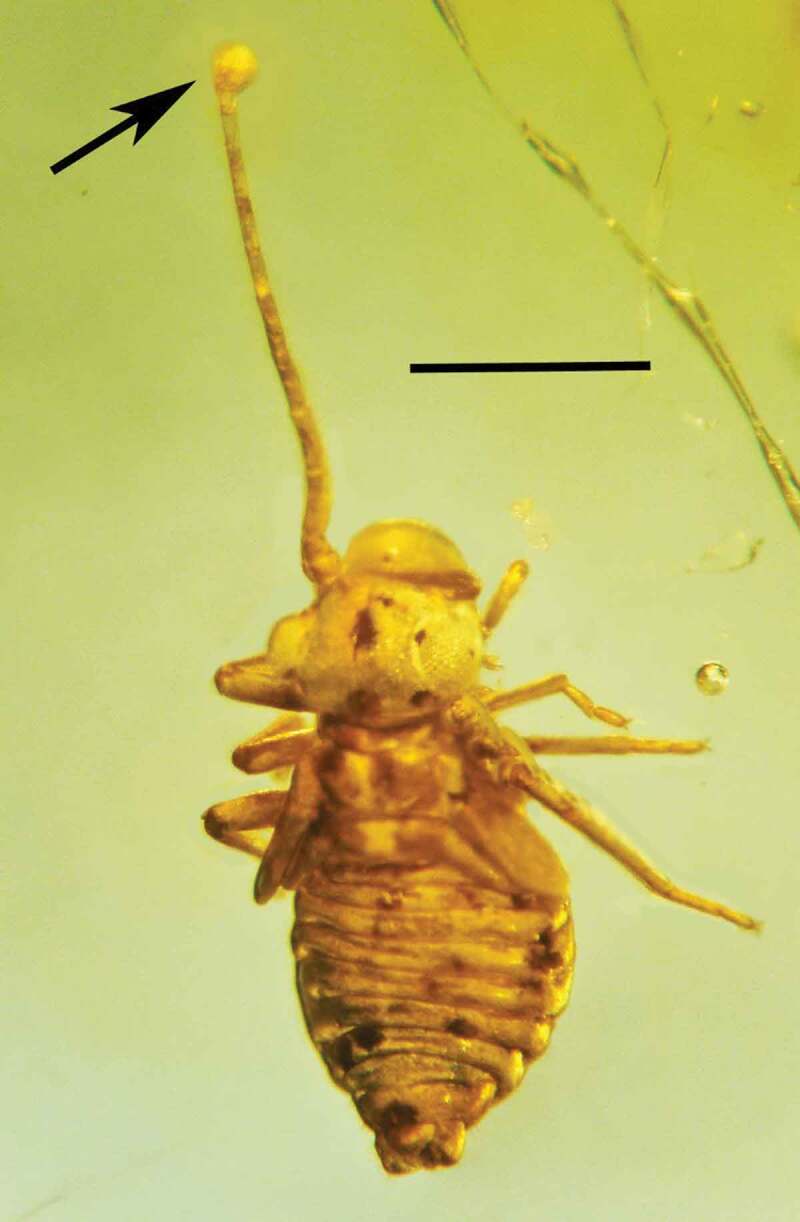
Dorsal view of the synnema (arrow) of *Polycephalomyces baltica* sp. nov. attached to the tip of the left antenna of a bark louse in Baltic amber. Bar = 0.7 mm.

**Figure 8.**
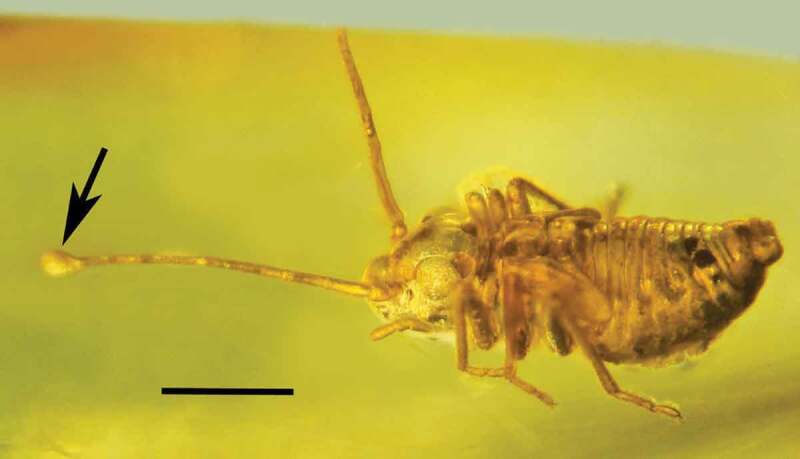
Lateral view of the synnema (arrow) of *Polycephalomyces baltica* sp. nov. attached to the tip of the left antenna of a bark louse in Baltic amber. Tip of right antenna was missing. Bar = 0.6 mm.

**Figure 9.**
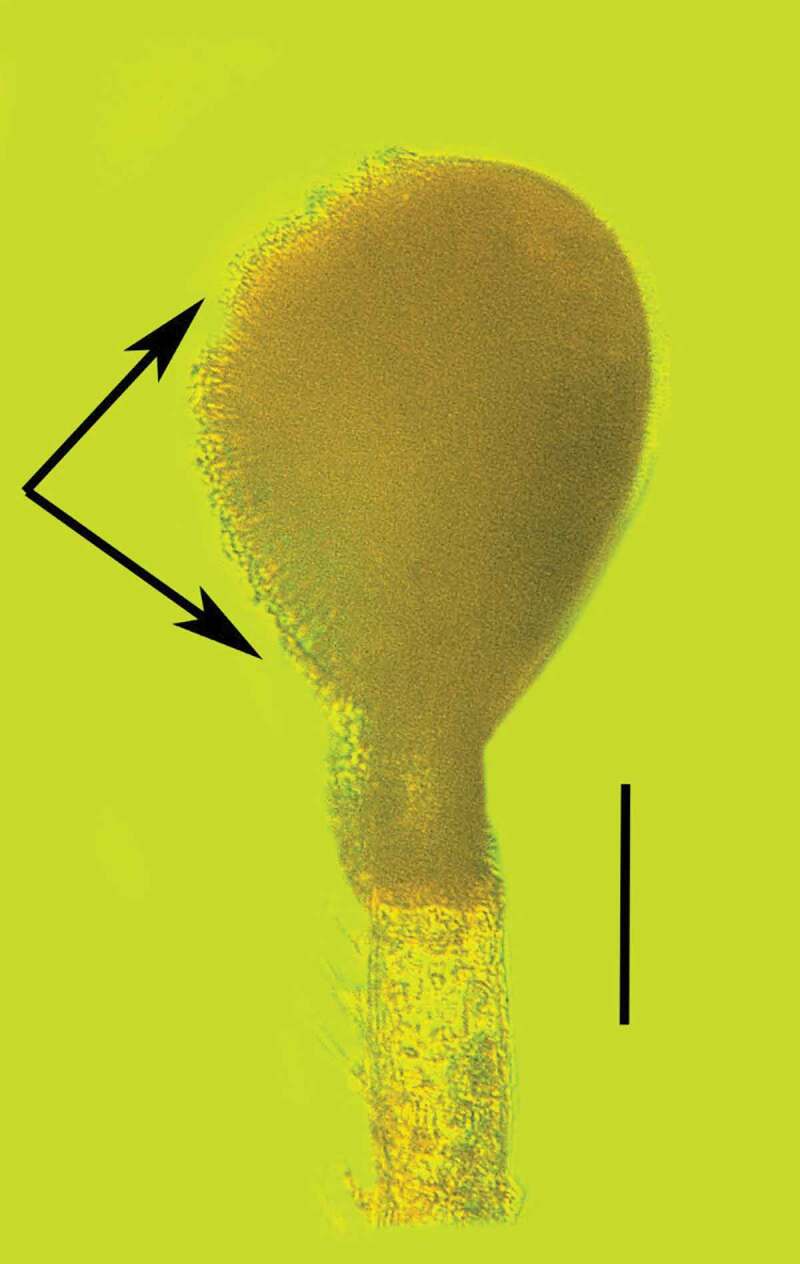
Synnema of *Polycephalomyces baltica* sp. nov. in Baltic amber showing conidiophores and conidia on left side (between the arrows). Arrowhead shows infected terminal antennomere of host. Bar = 78 µm.

**Figure 10.**
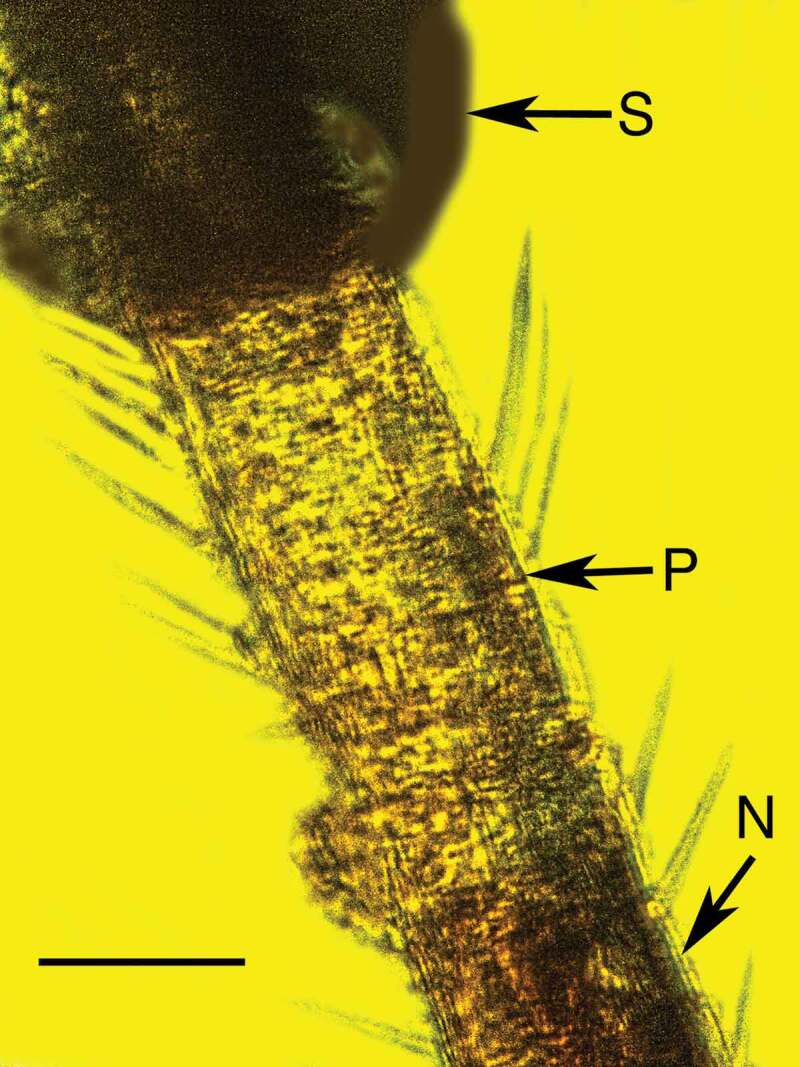
Infected terminal antennomere (P) of bark louse host subtending the synnema (S) of *Polycephalomyces baltica* sp. nov. in Baltic amber. N = uninfected antennomere. Bar = 84 µm.

**Figure 11.**
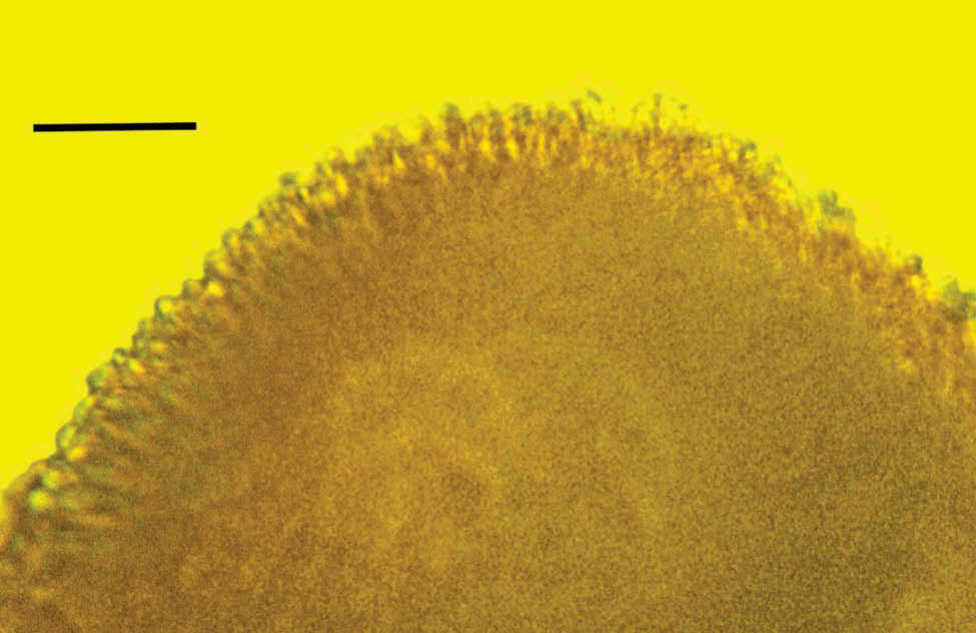
Portion of synnema of *Polycephalomyces baltica* sp. nov. showing conidiophores bearing developing conidia in Baltic amber. Bar = 20 µm.

**Figure 12.**
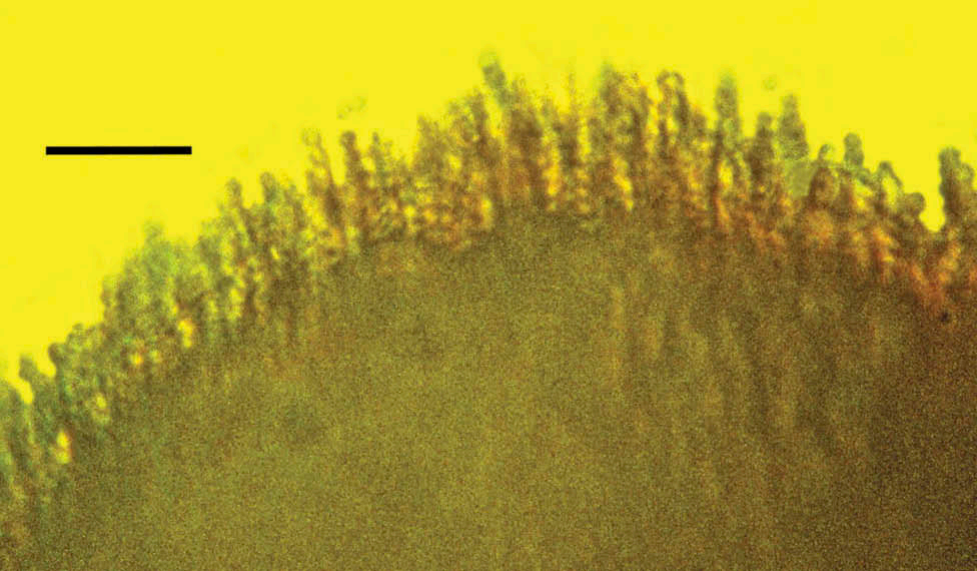
Detail of synnema of *Polycephalomyces baltica* sp. nov. showing conidiophores, phialides and catenulate, globose conidia in Baltic amber. Bar = 14 µm.

Type species: *Polycephalomyces baltica* Poinar & Vega sp. nov.*MycoBank*: 830,886*Polycephalomyces baltica* Poinar & Vega sp. nov. (Figures 7–12).

#### Diagnosis

Synnema small, roundish, light, borne at tip of host’s antenna; entire surface of synnema covered with unbranched chains of spores; conidiophores micronematous, phialides flask-shaped, diverging; conidia hyaline, globose, lacking surface ornamentation, with basipetal formation.

#### Description

Synnema simple, small, roundish, 170 µm in length, 142 µm in width, borne at tip of antennomere of bark louse (Psocoptera); aerial hyphae rare; entire surface of synnema covered with developing conidia; conidiogenous cells phialiform; conidiophores light coloured, micronematous, length 5–6 µm; phialides light coloured, micronematous, flask-shaped, non-diverging, length, 3–4 µm; conidia globose, catenulate, one-celled, hyaline, lacking surface ornamentation or mucus; basopetelate in unbranched spore-chains (with up to five spores in a chain), diameter of conidia, 2–3 µm.

##### Holotype

Holotype No. Sy-1-13B deposited in the Poinar amber collection maintained at Oregon State University.

#### Host

Nymph or short-winged female bark louse (Psocoptera: Troctopsocidae) (length, 1.9 mm).

#### Type locality

Samland Peninsula of the Baltic Sea in the Kalinin District of the Russian Federation.

#### Comments

While the Baltic amber specimen is tentatively assigned to the genus *Polycephalomyces*, without molecular data, it is not possible to determine its true affiliations within the Ophiocordycipitaceae. *Polycephalomyces baltica* sp. nov. is unique among entomopathogenic fungi by forming a rounded synnema bearing spores over its entire surface on the tip of the antenna of a bark louse. The shape of the synnema, its age and location on the terminal antennomere of a bark louse plus the nature of the phialides and conidia separate the fossil from other *Polycephalomyces* species (Kepler et al. 2013; Sanjuan et al. 2015).

In earlier arthropod-infecting anamorph genera that form synnemata, such as *Hymenostilbe, Isaria, Akanthomyces, Hirsutella*, and *Pseudogibellula*, the combined features of globose, hyaline, catenulate conidia formed in round, hyaline synnemata, parasitising bark lice, as found in *Polycephalomyces baltica*, are unknown (Barnett 1958; Madelin 1963; Kendrick and Carmichael 1973; Poinar and Thomas 1984).

## Discussion

The clavicipitoid fungi consist of three hypocrealean families: Clavicipitaceae sensu stricto, Cordycipitaceae and the Ophiocordycipitaceae. Members of the genus *Ophiocordyceps* in the Ophiocordycipitaceae include fungi that produce a darkly coloured “rigid, pliant or wiry stipe” and on hosts in soil or rotting wood (Kepler et al. 2013). Such a fungal and host pattern fits that of bark lice that occur on the bark of living or dying trees.

The Ophiocordycipitaceae parasitise a diverse clade of arthropods, including Coleoptera, Hemiptera, Hymenoptera and Lepidoptera (Sung et al. 2008; Kepler et al. 2013). The genus *Ophiocordyceps* sensu Petch, known to contain a diverse assemblage of Neotropical species, is characterised by clavate asci with gradually thickening apices and elongate, fusiform ascospores that do not articulate into part spores (Sanjuan et al. 2015). Species of this genus have been described as both sexual (teleomorph) and asexual (anamorph) species (Kepler et al. 2013; Sanjuan et al. 2015).

*Polycephalomyces* is characterised by superficial perithecia or perithecia immersed in an apical or subapical pulvinate cushion and long asci with ascospores forming many small part-spores of nearly equal length (Kepler et al. 2013). However, the genus has been used for both anamorph and teleomorph forms of some fungal clades (Kepler et al. 2013). The hosts thus far include nymphs of Cicadidae (Hemiptera), Neuroptera and larvae of Coleoptera (Kepler et al. 2013; Sanjuan et al. 2015). The bark louse host could be used as a diagnostic feature of *Polycephalomyces baltica.*

The conidia of *Polycephalomyces baltica* Poinar & Vega sp. nov. obviously penetrated the wall of the antennal segment to initiate infection. The antennal cell just below the synnema is mostly clear with some hyphal remains (Figure 10) and was probably the site of infection. The earliest fossil record of a synnema-producing entomopathogenic fungus is *Paleoophiocordyceps coccophagus* (Ophiocordycipitaceae) that was parasitising an Early Cretaceous scale insect in Burmese amber (Upper Albian: 97–105 Mya) (Sung et al. 2008).

Bark lice are known to be infected by representatives of several fungal lineages, including members of the genus *Conidiobolus* (Ancylistaceae: Entomophthorales) in Switzerland (Keller 2008), by unidentified fungi in Australia (Smithers 1972) and by *Pandora* (Zygomycota: Entomophthorales) and *Hirsutella* spp. in Argentina (Toledo et al. 2008, 2013). The records of *Hirsutella* spp. are the only other reports of members of the Hypocreales attacking these hosts (Toledo et al. 2008, 2013).

## Conclusions

These fossils represent the first fossil records of fungal infections of bark lice (Psocoptera). Both the Dominican amber species, *Ophiocordyceps dominicanus* Poinar & Vega sp. nov. and the Baltic amber species *Polycephalomyces baltica* Poinar & Vega sp. nov. represent extinct lineages and provide new characters (tubular synnema with a straight, pointed tip bearing spores over the entire surface and globular synnema forming on host’s antenna, respectively) that add to our understanding of the evolution of the entomopathogenic fungal representatives of the Hypocreales.
